# Progress in Obstetrics

**Published:** 1901-03-02

**Authors:** 


### PROGRESS IN OBSTETRICS.

**Tumours complicating Pregnancy.**—Dr. Gemmell[1] reports two cases of fibroid tumour complicating pregnancy, in both operation was performed and the patients recovered. In the one case the patient in addition to the tumour was suffering from eclampsia, sapraemia, and septic peritonitis. The abdomen was opened and a fibroid attached to the fundus of the uterus by a broad pedicle was discovered: it was lying retroverted in the pelvis, where it was adherent by recent peritonitis. Baer's operation was performed, the stump of the cervix being covered with peritoneum. Drainage was secured by means of an opening into the vagina, which was packed with gauze, and a glass tube in the lower angle of the abdominal incision. In the second case the patient was three months pregnant, and the tumour growing from the posterior wall of the uterus, completely blocking the pelvis and causing retention of urine. The abdomen was opened and the tumour with two smaller growths enucleated. The pelvis was flushed with hot water and the abdomen closed without drainage. Convalescence was rapid, and the patient went on to full term without inconvenience. Dr. Jardine[2] reports eight cases of labour complicated by tumours. In one in which there was a suppurating dermoid of the left ovary, and obstruction of the bowels from adhesions, the patient had been in labour four days, and at the time of operation was in a very collapsed and hopeless condition. The os was partly dilated, and the vertex presenting, the child was turned and delivered. Two hours after delivery the abdomen was opened, and the dermoid found to be attached to the bowel by firm adhesions; the cyst was removed and the abdomen well flushed with normal saline solution; but the patient never rallied, and died six hours after the operation. In five other cases Dr. Jardine was able to deliver by forceps or turning without interfering with the tumours. In another case the whole of the vulva was covered with large warty growths, evidently gonorrhœal, and very septic. After cleansing them as thoroughly as possible and plugging the vagina the growths were removed. When the os was sufficiently dilated the fœtus was delivered by turning. The patient made an uneventful recovery, the temperature remaining normal throughout. Dr. Amand Routh[3] reports a case of fibroids obstructing labour in which he performed Porro-Cæsarian hysterectomy instead of the Sänger-Cæsarian operation. The abdomen was opened in the middle line, the uterus incised, the child extracted, and the placenta and membranes removed. The uterus was then brought out through the abdominal incision, and the operation completed by Baer's method. Both mother and child had an uneventful recovery. Eugene R. Lewis[4] reports a case of myomectomy during the sixth month of pregnancy. The patient was a primipara 27 years of age. The tumour was as large as an orange, embedded in and projecting from the uterine wall. An incision was made in the uterus over the tumour, which was shelled out. The free hæmorrhage was controlled by very deep sutures. The patient made an uninterrupted recovery, and was delivered of a healthy child 3½ months after the operation. Lewis S. McMurtry[5] sums up the indications for treatment in cases of fibroid tumours complicating pregnancy as follows: Tumours of the fundus unless very large do not as a rule demand operation; tumours of the lower segment, causing pressure and pain and offering serious obstacles to delivery, must be removed. Hysteromyomectomy is the safest operation unless the pregnancy has advanced to almost full term, when a Porro-Cæsarian section may save both mother and child.

**Medullary Narcosis during Labour.**—S. Marx[6] reports several cases of lumbar cocainisation employed to lessen the pain of parturition. He gives the details of a number of cases in which this procedure was carried out, and says that although the cases are too few to warrant any wide deductions as to its ultimate value, he thinks that in lumbar cocainisation we have a method which is of the greatest value in producing anæsthesia, and almost abolishing the pain associated with labour, and that without danger to the mother or child. The puncture is made between the third and fourth, or fourth and fifth, lumbar vertebræ, the point of the needle being pushed slowly and gently downwards until cerebro-spinal fluid is seen to escape. From ten to fifteen minims of a 2 per cent. solution of cocaine are then injected. Within from two to fifteen minutes the anæsthesia commences, and it lasts from one to five hours. Uterine contractions go on regularly as if no narcotic had been used, the patient feeling no pain. There are two possible immediate dangers: (1) collapse from cocaine poisoning, (2) sepsis. Professor E. Bumm[7] reports six cases in which one centigramme of cocaine injected under the spinal meninges, between the fourth and fifth lumbar vertebræ, produced within from five to ten minutes complete analgesia of the lower extremities, extending to the sterno-costal arch, without, however, impairing the tactile sense, or suspending the control of the will over muscular action. Uterine contraction, on the other hand, continued as before, and was painless. Voluntary bearing down, moreover, proved as efficient as before the injection of cocaine. Efficiency of uterine contraction during the third stage remained unaffected, and primary perinæorraphy was done without pain. No serious complications occurred after the injection, although headache and vomiting were common.

**Puerperal Eclampsia.**—John F. Mosan,[8] writing on this subject, espouses the auto-intoxication theory. The kidney, he says, is the normal channel of escape, and so long as the renal functions are intact, the toxin can and usually does escape without doing harm. While the nature and origin of the toxin is not known, the liver is usually at fault. The toxin may be absorbed directly from the intestine, and if for any reason the liver is unable to deal with it, the excess will fall directly upon the kidney, probably injuring its finer structures and perhaps inflicting serious and permanent damage to the kidney itself. In cases of slight intoxication he recommends free purgation, preferably by mercurials, followed by salines, hot baths, milk diet, and rest in bed. As to the treatment of actual eclampsia he considers chloroform the remedy *par excellence* for controlling the convulsions. He also favours the use of morphia, ½ to 2 grains in 24 hours. Chloral is also favoured, but he condemns the use of pilocarpine. William Collier[9] draws attention to the danger incurred by the use of pilocarpine in puerperal eclampsia when the patient is unconscious. Pilocarpine not only causes profuse perspiration and salivation, but also an enormous increase in the mucus in the bronchial tubes, so that the unconscious patient runs the risk of being suffocated by her own secretions. Parak[10] explains his views on the nature of eclampsia by comparing the auto-intoxication of pregnancy with alcoholism. While chronic auto-intoxication is parallel to chronic alcoholism, eclampsia may be compared to drunkenness. Just as a man may die from acute or chronic alcoholic poisoning, so a woman may die of eclampsia, or more slowly from the effects of auto-intoxication upon the liver and kidneys. As regards treatment he recommends the following for the eclamptic seizure, inhalation of chloroform, lavage of the intestine, bleeding, and the subcutaneous injection of saline solution. Manipulation of the uterus is well known, he says, to inaugurate the convulsions; therefore he would avoid interference except when distinctly indicated in the interest of the mother and child, and then use chloroform pushed to the full surgical extent throughout the operation. W. A. Newman Dorland[11] believes the liver plays an important part in the production of eclampsia, and this theory of the hepatic origin of puerperal eclampsia affords ample explanation of those cases not associated with albuminuria. As to what the poisonous substance is that is directly responsible for the eclamptic seizure opinions are at variance. The products of metabolism in both fœtus and mother are carried to the maternal liver, where they normally undergo katabolic changes to urea and bile salts; but in cases of hepatic inadequacy these products accumulate in the blood and produce eclampsia. Dr. Krusen[12] says he firmly believes that if we used more salines and calomel during the last three months of pregnancy, and thus stimulate the hepatic activity, the causes of eclampsia might be eliminated.

[1] Lancet, Nov. 17th, 1900, J. E. Gemmel. [2] Glasgow Med. Journ., August 1900, Dr. Jardine. [3] Lancet, July 14th, 1900, Dr. Amand Routh. [4] Amer. Journ. of Surg. and Gynæcology, September 1900, E. R. Lewis. [5] New York Med. Journ., Sept. 1st, 1900, Lewis S. McMurtry. [6] Med. Rec., Oct. 6th, 1900, S. Marx. [7] The Med. Age, Sept. 25th, 1900, E. Bumm. [8] Amer. Journ. of Obstetrics, September 1900, J. F. Moran. [9] Brit. Med. Journ., Sept. 22nd, 1900, William Collier. [10] Med. Chron., November 1900, Parak. [11] Amer. Journ. of Obstetrics, September 1900, W. A. Newman Dorland. [12] Amer. Journ. of Obstetrics, September 1900, Dr. Krusen.

